# PLX4032, a selective BRAF^V600E^ kinase inhibitor, activates the ERK pathway and enhances cell migration and proliferation of BRAF^WT^ melanoma cells

**DOI:** 10.1111/j.1755-148X.2010.00685.x

**Published:** 2010-02-10

**Authors:** Ruth Halaban, Wengeng Zhang, Antonella Bacchiocchi, Elaine Cheng, Fabio Parisi, Stephan Ariyan, Michael Krauthammer, James P McCusker, Yuval Kluger, Mario Sznol

**Affiliations:** 1 Department of Dermatology, Yale University School of MedicineNew Haven, CT, USA; 2 Department of Cell Biology, New York University Center for Health Informatics and Bioinformatics, New York University School of Medicine and Cancer InstituteNew York, NY, USA; 3 Department of Surgery, Yale University School of MedicineNew Haven, CT, USA; 4 Department of Pathology, Yale University School of MedicineNew Haven, CT, USA; 5 Comprehensive Cancer Center Section of Medical Oncology, Yale University School of MedicineNew Haven, CT, USA

**Keywords:** BRAF, cell migration, drug response, ERK pathway, melanoma, RAF1, serum markers

## Abstract

BRAF^V600E/K^ is a frequent mutationally active tumor-specific kinase in melanomas that is currently targeted for therapy by the specific inhibitor PLX4032. Our studies with melanoma tumor cells that are BRAF^V600E/K^ and BRAF^WT^ showed that, paradoxically, while PLX4032 inhibited ERK1/2 in the highly sensitive BRAF^V600E/K^, it activated the pathway in the resistant BRAF^WT^ cells, via RAF1 activation, regardless of the status of mutations in NRAS or PTEN. The persistently active ERK1/2 triggered downstream effectors in BRAF^WT^ melanoma cells and induced changes in the expression of a wide-spectrum of genes associated with cell cycle control. Furthermore, PLX4032 increased the rate of proliferation of growth factor-dependent NRAS Q61L mutant primary melanoma cells, reduced cell adherence and increased mobility of cells from advanced lesions. The results suggest that the drug can confer an advantage to BRAF^WT^ primary and metastatic tumor cells in vivo and provide markers for monitoring clinical responses.

## Significance

The identification of druggable kinases in cancers is currently a promising approach for the development of patient-tailored therapy. However, tumors harbor various mutations in proliferation/survival pathways that can diminish drug efficacy. We report here studies on the effects of PLX4032 on short-term cultures of human melanoma cells that have been characterized for mutations in known genes. PLX4032 is a BRAF^V600K^ kinase inhibitor that has demonstrated encouraging responses in current Phase I/II clinical trials. We explored the mechanism by which non-responsive BRAF^WT^ melanoma cells escape inhibition and show that these cells are stimulated by the drug in ways that can confer growth advantage in vitro. Our results suggest that only patients with mutant BRAF-V600K/E should be selected for treatment and that patients should be monitored for any secondary tumors that may not carry the BRAF mutation, or for recurrences of tumor cells that have lost the mutant BRAF allele.

## Introduction

Tumor-specific activated kinases that confer uncontrolled cell proliferation to cancer cells and promote metastasis have been attractive targets for therapy, because cancer cells are often dependent on this class of molecule, a condition termed ‘oncogene addiction’, while normal cells are not (Sharma and Settleman, 2007; Weinstein and Joe, 2008). Consistent with this paradigm are the clinical successes with the multi-kinase inhibitor imatinib (Gleevec) in treating chronic myelogenous leukemia and gastrointestinal stromal tumors, cancers dependent on the ABL kinase and the receptor kinase c-KIT, respectively (Sharma and Settleman, 2007; Weinstein and Joe, 2008). Likewise, melanoma patients with activating mutations in c-Kit are also being treated with Imatinib (acral and mucosal melanomas) (Curtin et al., 2006; Dhomen and Marais, 2009; Hodi et al., 2008; Jiang et al., 2008), and those with activated BRAF, present in about 60% of cases (Forbes et al., 2008; Smalley et al., 2009), are currently selected for enrollment in phase I/II clinical trials with PLX4032, an inhibitor of activated BRAF^V600E^ that has generated promising outcomes (Flaherty et al., 2009 and http://www.news-medical.net/news/20090930/Plexxikon-commences-patient-dosage-in-PLX4032-trials-for-metastatic-melanoma.aspx).

Here, we assessed the effects of PLX4032 on freshly isolated cultured melanoma cells harboring different mutations and explored the mechanism by which non-responsive BRAF^WT^ melanoma cells escape drug inhibition. We demonstrate that, paradoxically, whereas PLX4032 inhibited extracellular signal-regulated kinase (ERK) in BRAF^V600E/K^-mutants, it induced the pathway in BRAF^WT^ melanoma cells via activation of RAF1. PLX4032 promoted the proliferation of growth factor-dependent, NRAS mutant, primary melanoma cells, reduced cell adhesion and increased cell motility of highly proliferating, mitogen- independent advanced melanoma cells.

## Results

### Growth responses of melanoma cells to PLX4032

**Figure 1:**
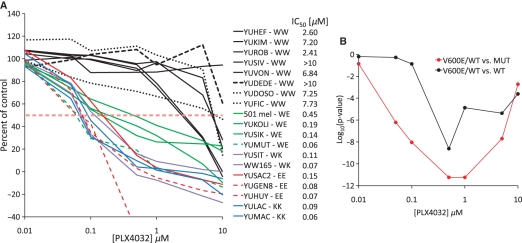
Growth responses to PLX4032. (A) CellTiter-Glo® Luminescent Cell Viability Assay. Values are percent of control (DMSO) assessed at the end of 72-h treatment with PLX4032 (1 μM). Each measurement is the average of triplicate or quadruplet wells. The legend on the right provides the IC_50_ indicating melanoma cells that are BRAF homozygous or heterozygous for wild-type (W), V600K (K) or V600E (E) mutation. Among the BRAF^WT^ cell strains, YUDOSO and YUFIC are heterozygous for the NRAS Q61K and Q61R mutation, respectively (dotted black line), and YUDEDE cells are null for PTEN and carry homozygous NRAS Q61H mutation (dashed black line). Green and red dashed lines indicate BRAF mutant cells null for PTEN. The horizontal dashed line marked IC_50_. STDV was about 5% of total count. (B) A two-sample Wilcoxon rank sum test assessing differences in growth responses of wild-type and mutant melanoma cells at several concentrations of PLX4032. The graphs, plotted as logarithm of the P-values (*y*-axis) over PLX4032 concentration show significant differences over a broad range of PLX4032 concentrations in the response of cells carrying the heterozygous BRAF^V600E/WT^ alleles compared to BRAF^WT^ melanoma cells (red), and BRAF^V600E/WT^ compared with other mutant cells, i.e. homozygous BRAF^V600E/E^, BRAF^V600K/K^ or heterozygous BRAF^V600K/WT^ (black).

The effect of PLX4032 was tested on melanoma cells isolated from primary and metastatic lesions in which BRAF, NRAS and PTEN mutations were characterized (Table 1). Dose response analyses showed that all the BRAF mutant melanoma cell strains were highly sensitive to PLX4032 with IC_50_ in the nM range (60–450 nM), whereas BRAF wild-type cells were resistant, with IC_50_ 2.4 μM or above (Figure 1A), consistent with published information (Tsai et al., 2008). Interestingly, three of four heterozygous V600E/WT cell strains (501 mel, YUKOLI and YUSIK) were slightly, but significantly more resistant compared with the other mutant cells (Figure 1A, green solid lines). The differences were statistically significant across a wide range of PLX4032 concentrations as shown by the two-sample Wilcoxon rank sum test (Figure 1B). The outlier, YUMUT-BRAF^V600E/WT^ melanoma cells, are also PTEN null and further examination is needed to establish whether mutations complementing the heterozygous V600E mutation confer more sensitivity to the drug. Different levels of BRAF or RAF1 (also known as c-RAF) proteins (Figure S1) could not explain the differences in growth responses to PLX4032. The results demonstrated that drug response can be modulated by the BRAF genotype but is not affected by mutations in NRAS or downregulation of PTEN in BRAF^WT^ melanoma cells isolated from advanced lesions.

**Table 1:** Sources of patient’s derived melanoma cells and their mutations status

Melanoma	Gender/age	Stage/site	BRAF/NRAS mutation	PTEN
Wild-type for BRAF and NRAS				
YUHEF	M/52	IV, lung	WT	WT
YUVON	M/63	IV, acral	WT	WT
YUROB	F/76	II, abdominal wall	WT	LOH
YUSIV	F/61	IV, chest	WT	WT
YUKIM	F/71	III, lymph node	WT	WT, LOH
BRAF mutants/NRAS wild-type				
YUMAC	M/68	IV, soft tissue, thigh	V600K	WT
YUGEN8	F/44	IV, brain	V600E	Null
YUHUY	M/64	II, lymph node	V600E	Null
YUSAC2	M/57	IV, soft tissue, neck	V600E	WT/LOH
YULAC	F/66	IV, soft tissue, neck	V600K	P38S/LOH (C1143T)
YURIF	M/52	IV, soft tissue, thigh	V600K	WT
WW165	F/62	Primary melanoma, 2.25 mm	V600K/WT	WT
YUKOLI	M/53	IV, lymph node	V600E/WT	WT
YUSIT1	M/67	IV, unknown	V600K/WT	WT
YUSIK	F/49	IV, lymph node	V600E/WT	WT
501 mel^a^	Not known	IV, lymph node	V600E/WT	WT
YUMUT	M/44	III, soft tissue, wrist	V600E/WT	Null
NRAS mutants/BRAF wild-type				
YULOVY	F/83	I, primary melanoma, right medial calf, 1.38 mm	Q61L/WT	WT
YUDOSO	M/84	llb, primary melanoma, left abdomen, 2.5 mm	Q61K/WT	WT
YUDEDE	M/83	III, soft tissue, pretibial	Q61H	Null
YUFIC	M/65	IV, lymph node	Q61R/WT	WT
YUFULO	M/87	Primary, choroid	Q61L/WT	ND

ND, not determined.

a The melanoma cell 501 mel carries also activating S33C mutations in mutation in β-catenin (Halaban et al., 2009).

### PLX4032 activates ERK in BRAF^WT^ melanoma cells

**Figure 2:**
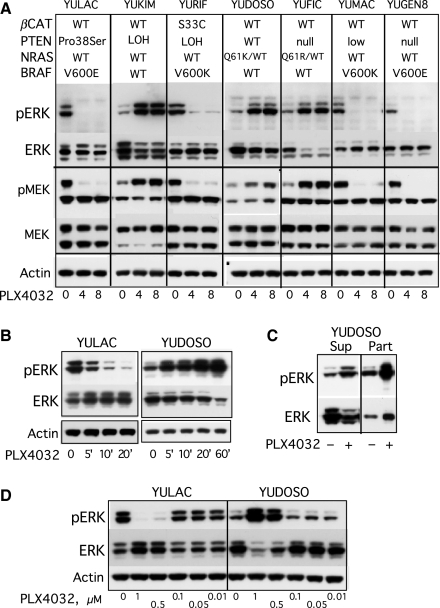
Changes in ERK1/2 and MEK in response to PLX4032. (A) Melanoma cell strains were treated with DMSO for 4 h (0), or with PLX4032 (1 μM) for 4 and 8 h. The panels show Western blots probed with antibodies to phosph-ERK1/2 Thr202/Tyr204 mAb (pERK), ERK1/2 (ERK), phospho-MEK1/2 (pMEK), MEK1/2 (MEK), and actin as a loading control. The mutation status of BRAF, NRAS, PTEN and β-catenin (βCAT) are indicated at the top. (B) Western blot analyses of ERK1/2 inactivation/activation after short-term incubation with PLX4032 (1 μM). (C) pERK1/2 and ERK1/2 in supernatant (Sup) and particulate (Part) fractions. (D) Changes in pERK1/2 activation after treatments with increasing concentration of PLX4032, or DMSO for 1 h.

The effects of PLX4032 on downstream RAF effectors were examined to further understand the mechanism of drug resistance. Unless otherwise stated, we used 1 μM of PLX4032, about 10× the IC_50_ of sensitive melanoma cells, and equal amounts of the solvent DMSO (0.1%) as a control. Consistent with published data (Sala et al., 2008; Tsai et al., 2008), PLX4032 abolished the ERK1/2 activating phosphorylation in BRAF^V600E/K^ melanoma cells (Figure 2, pERK, YULAC, YURIF, YUMAC and YUGEN8). However, unlike published reports, PLX4032 induced ERK1/2 phosphorylation in BRAF^WT^ melanoma cells (Figure 2, pERK, YUKIM, YUDOSO and YUFIC). Increased ERK1/2 phosphorylation in these cells was persistent and was independent from the status of PTEN (compare YUDOSO with YUFIC), or the presence of NRAS activating mutation (compare the NRAS wild-type YUKIM with NRAS mutant YUDOSO and YUFIC cells). In addition, mutationally active β-catenin did not diminish the effect of PLX4032 on BRAF^V600K^ mutant melanoma cells (Figure 2, YURIF). Examination of MEK, the upstream activator of ERK, showed a similar pattern of inactivation and activation in response to PLX4032 (Figure 2, pMEK), demonstrating that while the RAF-MEK-ERK pathway was inhibited in BRAF mutants, it was activated in BRAF^wt^ melanoma cells.

Changes in dephosphorylation and hyperphosphorylation of ERK1/2 in YULAC-BRAF^V600E^ and YUDOSO-BRAF^WT^ melanoma cells, respectively, occurred within 5 min, and progressed with similar kinetics (Figure 2B, pERK). The Western blots also showed that the levels of total ERK1/2 protein in BRAF^WT^ cell lysates decreased after treatment with PLX4032 (Figure 2A, ERK, YUKIM, YUDOSO and YUFIC, compare 0 to 4 and 8 h drug treatment, and Figure 2B, YUDOSO), although actin levels were the same (Figure 2A, B, actin). Because activated ERK1/2 translocates to the nucleus and might have remained RIPA insoluble, we examined the particulate fractions after solubilization in SDS-sample buffer, heating and sonication. The results show enrichment of phospho-ERK1/2 and total ERK1/2 in the particulate compartment of YUDOSO-BRAF^WT^ after treatment with PLX4032 (Figure 2C, compare lanes marked Sup and Part).

The opposite effects of PLX4032 on ERK1/2 phosphorylation in YULAC-BRAF^V600E^ and YUDOSO-BRAF^WT^ melanoma cells were concentration dependent. Both cell types responded to the drug at 1 and 0.5 μM, but not at 0.1 μM (Figure 2D), in good agreement with the known IC_50_ of PLX4032 against purified BRAF^V600E^ kinase (44 nmol/l) (Sala et al., 2008).

Other intracellular signaling pathways were not, or slightly affected by PLX4032. We did not detect engagement of the AKT pathway (Figure S2A, pS6K and pAKT). There were only slight changes in the activated form of p38MAPK in YULAC-BRAF^V600E^ and YUDOSO-BRAF^WT^ in response to the drug (Figure S2B). Although the levels of phospho JNK T183/Y185 were induced within 30 min of PLX4032 treatment (Figure S2C), there were no changes in the activated status of several known downstream JNK targets, such as p53, JUN and eIF4E in YUDOSO-BRAF^WT^ melanoma cells, whereas only phospho-eIF4E Ser209 levels were reduced in YULAC-BRAF^V600E^ cells (Figure S2D), suggesting very little functional consequences of JNK activation on BRAF^WT^ melanoma cells. We therefore focused our studies on the ERK pathway.

### PLX4032 activates RAF1 in BRAF^WT^ melanoma cells

We ruled out ERK activation by MEKK1 (Lu et al., 2002) because YUKIM melanoma cells in which ERK1/2 was highly activated in response to PLX4032 (Figure 2A) did not express the protein (data not shown). We also ruled out MEKK3 (Craig et al., 2008), because this enzyme was inhibited by PLX4032 in YUDOSO-BRAF^WT^ (Figure S4). Suppression of two dual-specificity phosphatases, MKP-1 and MKP5 was also an unlikely mechanism, because the two remained unchanged in YUDOSO-BRAF^WT^ cells after treatment with PLX4032 (data not shown).

**Figure 3:**
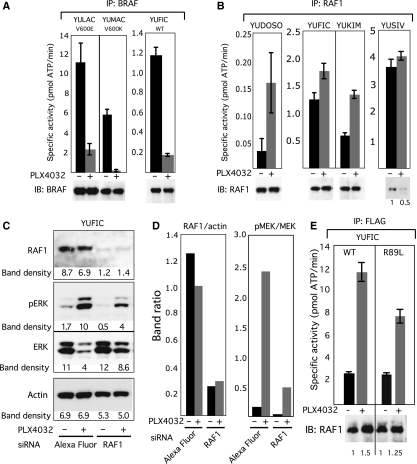
RAF kinase in response to PLX4032. (A, B, E) Immune-complex kinase activities expressed as picomoles [γ-^32^P]-ATP incorporated into MBP. Each data point is an average of triplicate measurements ± STDV. The cells were treated with PLX4032 for 1 h (A), 4 h (B) and 16 h (E). The panels under each kinase assay shows Western blots of immunoprecipitated proteins eluted with SDS sample buffer and probed with antibodies to the respective protein. Numbers under the protein bands in (B) and (E) indicate relative band density determined by densitometric analysis. (C) Suppression of PLX4032 induced ERK activation in YUFIC-BRAF^WT^ melanoma after RAF1 depletion by siRNA. YUFIC melanoma cells were transfected with Alexa Fluor or RAF1 siRNA and 2 days later were treated with PLX4032 or DMSO for 1 h. Cell lysates were probed with the indicated antibodies. The numbers under each blot represent values of band density in pixels 1 × 10^3^. (D) Histograms showing band intensity ratios normalizing RAF1 to actin and pERK to total ERK as presented in (C).

We therefore assessed BRAF and RAF1 enzymatic activity. Immune-complexes kinase assays showed, as expected, high BRAF activity in YULAC-BRAF^V600E^ and YUMAC-BRAF^V600K^ cells that was suppressed after treatment with PLX4032 for 30 min (Figure 3A, YULAC and YUMAC). In BRAF^WT^ melanoma cells, BRAF was either not detectable or was about 10- to 50-fold less active than its mutant counterpart. In cases when activity was measurable, PLX4032 inhibited the wild-type BRAF kinase as well (Figure 3A, YUFIC).

Similar studies with RAF1 showed non-detectable activity in YULAC-BRAF^V600E^ and YUMAC-BRAF^V600K^ cells (data not shown). In contrast, a wide range of RAF1 kinase activity was observed in four independent BRAF^WT^ melanoma cells (0.03–3.7 pmol ATP/min), that in all cases was further increased after treatments with PLX4032 (Figure 3B). Furthermore, fivefold reduction in RAF1 levels in YUFIC-BRAF^WT^ melanoma cells by siRNA (Figure 3C, compare RAF1 bands with actin as the loading control, and Figure 3D, RAF1/actin ratio) caused a similar fold suppression in PLX4032 induced ERK activation compared with non-treated cells (Figure 3C, pERK and ERK, and Figure 3D, pERK/ERK ratio), lending support to the conclusion that RAF1 is the main kinase that caused ERK activation.

We considered several known pathways by which PLX4032 could activate RAF1. We ruled out triggering an escape pathway, such as a receptor tyrosine kinase, by two independent approaches. First, traditional Western blotting with anti-RAF1 phospho-specific antibodies failed to detect an increase in RAF1(S338) or RAF1(S259) phosphorylation (Figure S3A, B). There was a persistent increase only in phospho-RAF1(Ser289/296/301), the ERK1/2 phosphorylation sites, in BRAF^WT^ cells treated with the drug (Figure S3A, B). However, the phosphorylation of these sites serves as a feedback mechanism to attenuate ERK1/2 activity (Baccarini, 2005) and therefore cannot explain RAF1 activation. Second, transiently expressed mutant RAF1 R89L, that does not bind Ras-GTP, a critical step in receptor-mediated stimulation, was activated by PLX4032 to the same extent as ectopically expressed wild-type RAF1 (Figure 3E). We therefore concluded that PLX4032 did not activate an upstream RAF1 kinase escape pathway.

Another major mechanism for RAF1 activation is homodimerization or heterodimerization with wild-type or enzymatically impaired mutant BRAF (Garnett et al., 2005; Heidorn et al., 2010; Rajakulendran et al., 2009; Rushworth et al., 2006). However, we could not demonstrate co-immunoprecipitation of endogenous BRAF with RAF1, or transiently transfected MYC and FLAG tagged RAF1 in PLX4032-treated melanoma cells under conditions in which RAF1 was activated by the drug.

### Differential activation of downstream ERK1 targets

**Figure 4:**
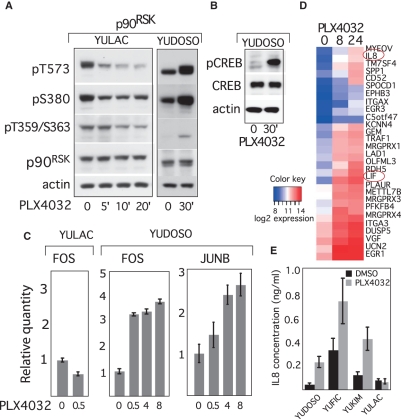
Inactivation/activation of downstream ERK targets in response to PLX4032. (A, B) Western blots showing changes in p90^RSK^ and CREB activation states, respectively, employing phospho-specific antibodies. (C) Changes in c-FOS and JUNB gene transcripts in response to PLX4032 as evaluated by Real Time RT-PCR. Data are averages of three replicates ± STDV. (D) Heatmap showing upregulation of gene expression by at least 3-fold and above in YUDOSO-BRAF^WT^ melanoma cells in response to treatment with PLX4032 for 8 and 24 h employing the NimbleGen whole genome expression arrays. Marked in red circles are IL8 and LIF. (E) ELISA assay confirming an increase in secreted IL8 levels after 24 h incubation with PLX4032 in BRAF^WT^ (YUDOSO, YUFIC and YUKIM), but not in mutant melanoma cells (YULAC). Error bars represent STDV of six wells.

We further explored the activation of downstream ERK targets and changes in gene expression that may shed more light on PLX4032 cellular responses and may provide markers to monitor therapy. Western blotting with phospho-specific p90^RSK^ antibodies revealed marked activation of this ERK1/2 effector in YUDOSO-BRAF^WT^ melanoma cells, while it was inhibited in YULAC-BRAF^V600E^ (Figure 4A, p90^RSK^, T573, S380 and T359/S36). The nuclear resident transcription factor CREB, a known p90^RSK^ downstream target (21) was also activated (Figure 4B).

Real time RT-PCR demonstrated that known early response genes, FOS and JUNB, were activated within 30 min in YUDOSO-BRAF^WT^ melanoma cells in response to PLX4032, an effect that was persistent for up to 8 h, whereas FOS was downregulated within 30 min in YULAC-BRAF^V600E^ cells (Figure 4C), in agreement with the kinetics of ERK1/2 functional activation and inactivation in wild-type and mutant cells, respectively.

We explored the spectrum of affected genes by hybridization to NimbleGen whole genome gene expression arrays, comparing untreated to PLX4032 treated (8 and 24 h) YUDOSO-BRAF^WT^ melanoma cells. The results showed strong upregulation (3-fold and more) of 28 genes in response to PLX4032, including the early response genes EGR1 and EGR3 (Figure 4D). Of interest is the persistent activation of IL8 and LIF by 6.3- and 12.6-fold, respectively (Figure 4D, marked with red circles), because these two are secreted proteins that may serve as markers for ERK activation. Indeed, ELISA tests confirmed that PLX4032 activated IL8, showing that the protein was secreted into the medium not only in YUDOSO, but also in two additional BRAF^WT^ melanoma cells YUKIM and YUFIC, while it was not activated in YULAC BRAF^V600E^ cells (Figure 4E).

Gene set enrichment analysis (GSEA) demonstrated upregulation of downstream targets of EGR1, ATF2 and CREB with a FDR (false discovery rate) of less then 25%, consistent with CREB activation (Figure 4B). GSEA pathway analysis featured a ‘Ras Oncogenic Signature’ (Bild et al., 2006) and TGFB as the top gene sets with a FDR of less then 5%. The full list of PLX4032 induced changed genes are provided in Table S2 and will be uploaded on the GEO profile site.

### Functional responses to PLX4032

One of the main questions raised by our studies is whether ERK activation had any impact on cellular functions, because we did not detect a significant increase in the proliferation rate of advanced BRAF^WT^ melanoma cells treated with PLX4032. We reasoned that these cells could not be further stimulated because they were proliferating already at their maximal rate, but cells from early stage melanoma that did not gain full independence from external growth factors required by normal melanocytes (Böhm et al., 1995), may respond differently. To explore this notion, we examined the effect of PLX4032 on primary melanoma cells, YULOVY isolated from a 1.38 mm primary cutaneous lesion and YUFULO ocular melanoma, both harboring the NRAS Q61L mutation, compared with melanocytes from a giant nevus (YUREEL-NV) and keratinocytes, all wild-type for BRAF and NRAS.

**Figure 5:**
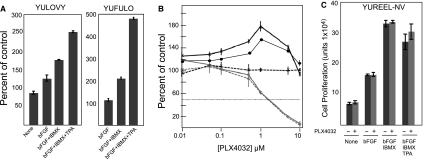
Proliferation of mitogen-dependent cells in response to PLX4032. (A) YULOVY and YUFULO cell proliferation after incubation for 72 h in basal medium (none) or medium supplemented with growth factors as indicated. Values are averages of triplicate wells expressed as percent of control (Day 0). (B) Dose response to increasing concentrations of PLX4032. YULOVY and YUFULO, solid dark lines with X and solid circles, respectively, grown in medium supplemented with bFGF and IBMX; YULOVY cells grown with bFGF and IBMX, broken dark line; Keratinocytes grown without and with supplements, gray solid and broken lines, respectively. Other details as in Figure 1. (C) YUREEL-NV melanocyte proliferation in basal medium without growth factors (none), and with growth factors (as indicated) in the absence and presence of PLX4032. Error bars represent STDV of triplicate wells.

In agreement with this concept, YULOVY and YUFULO cells that require bFGF, IBMX and TPA for optimal proliferation (Figure 5A), were stimulated by PLX4032 when incubated in suboptimal conditions, i.e. with bFGF and IBMX (Figure 5B, solid dark lines), but not in the full spectrum of required growth factors (Figure 5B, broken dark line). In contrast, the keratinocytes were inhibited in the presence or absence of growth supplements, with a similar IC_50_ of 1.54 and 1.6 μM, respectively (Figure 5B, solid and broken gray lines). Interestingly, YUREEL-NV melanocytes that are wild-type for BRAF and NRAS, and also mitogen-dependent, were not affected under any conditions (Figure 5C). It should be noted that during this 72 h test, the keratinocytes’ growth rate was the same whether or not supplements were provided.

**Figure 6:**
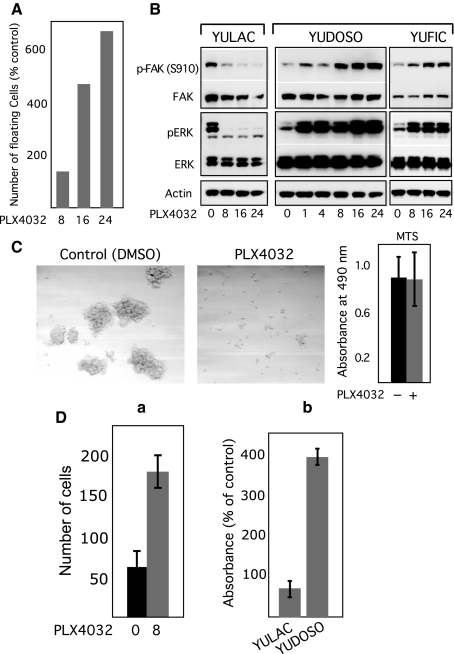
PLX4032 reduced cell adhesion and promoted migration in BRAF^WT^ melanoma cells. (A) The histograms show the number of floating YUDOSO-BRAF^WT^ melanoma cells at different time intervals (in hours) after the addition of PLX4032 as percent of control (2500 floating cells). (B) Changes in FAK activation in response to PLX4032, as detected by Western blotting with antibodies against phospho FAK (S910), FAK, phospho-ERK1/2, ERK1/2 and actin. (C) Low magnification photomicrographs of 10 days soft agar cultures showing YUDOSO-BRAF^WT^ cells forming large colonies in control (DMSO), but single cells or small colonies in PLX4032. The histogram demonstrates the viability (MTS) of cells in 96-well soft agar plates measuring absorbance at 490 nm. (D) Cell migration in response to PLX4032. The histograms show the number of cells that migrated thorough the transwell pores by counting three microscopical fields after 8 h treatment (a), or by extracting the stained cells and measuring absorbance at 570 nm after 24 h incubation with the drug normalized to control, DMSO treated cells (b).

PLX4032 also had physiological effects on advanced melanoma cells. We observed enhanced detachment of BRAF^WT^ melanoma cells after treatment with PLX4032 for several hours that were 99% viable (Figure 6A). In contrast, the number of YULAC-BRAF^V600E^ floating cells did not change under similar conditions (about 700 cells in 60 mm Petri dish seeded at 90% confluency). This led us to assess focal adhesion kinase (FAK) activation and changes in cell adhesion and migration (Mitra et al., 2005). The results showed an increase in phospho-FAK (S910), the ERK1/2 phosphorylation site, in YUDOSO-BRAF^WT^ and a reduction in YULAC-BRAF^V600E^ melanoma cells (Figure 6B, pFAK S910). Although there was a slight increase in phospho-FAK S910 within 1 h, the major activation occurred 8 h later and was maintained at high levels further on (Figure 6B, compare pFAK S910 with pERK1/2), suggesting an intermediary limiting step.

Two additional assays confirmed that activated FAK had a functional impact on BRAF^WT^ melanoma cells. First, there was a dramatic reduction in colony formation in soft agar in response to PLX4032 (Figure 6C), although the number of viable cells was similar (Figure 6C, MTS). Second, the transwell-based migration assay showed that PLX4032 enhanced the motility of YUDOSO-BRAF^WT^, but not that of YULAC-BRAF^V600E^ melanoma cells (Figure 6D(a, b)). There was approximately fourfold increase in YUDOSO-BRAF^WT^ migrating cells compared with control, whereas the number of migrating YULAC-BRAF^V600E^ melanoma cells was reduced by 30% (Figure 6D(b)). In contrast, PLX4032 did not affect cell invasion, because very few YUDOSO-BRAF^WT^ cells invaded through the Matrigel after 24, 48, and 72-h incubation in the absence or presence of PLX4032 (data not shown).

## Discussion

In the studies described here, we employed cultured melanoma cells freshly isolated from individual patients’ tumors as well as normal skin cells to investigate the impact of genetic variations on current therapy with PLX4032. We demonstrated that while PLX4032 inhibited ERK1/2 in BRAF^V600E/K^, it activated this signaling pathway in BRAF^WT^ melanoma cells via stimulation of RAF1 in a RAS-independent manner. Activating mutations in NRAS and β-catenin, or loss of PTEN did not affect the responses of BRAF^WT^ melanoma cells to this BRAF inhibitor. PLX4032 enhanced the rate of proliferation of mitogen-dependent primary melanoma cells carrying the NRAS Q61L mutation, and decreased adhesion and increased migration, of rapidly dividing melanoma cells from advanced lesions, changes that may confer tumor advantage in vivo. Interestingly, while the proliferation of benign melanocytes isolated from a giant nevus was not affected, the drug inhibited keratinocytes. The latter results are not in contradiction with in vivo observations, i.e. an increase in the incidence of cutaneous squamous cell carcinoma in patients chronically exposed to the drug (Flaherty et al., 2009), because the keratinocytes were isolated from basal and not squamous epithelium, which is composed of differentiated cells likely to have different growth properties. We also report for the first time inhibition of the ERK1/2 kinase MEKK3 in BRAF^WT^ cells treated with this PLX4032.

Activation of ERK1/2 by RAF inhibitors, such as SB-590885 and ZM 336372, has been reported before (Hall-Jackson et al., 1999; King et al., 2006), but the mechanism and consequences of such activation were not explored in these previous studies. In the course of peer review, two manuscripts were published that confirm our results (Hatzivassiliou et al., 2010; Heidorn et al., 2010). In these reports, the investigators also observed that selective BRAF inhibitors, such as PLX4720, 885-A and GDC-0879 stimulated MEK–ERK signaling in BRAF wild-type melanoma and carcinoma cells via RAF1 activation (Hatzivassiliou et al., 2010; Heidorn et al., 2010). Dr Marais and his collaborators went one step further, showing that mutationally inactive BRAF^D594A^ cooperates with oncogenic KRAS^K12D^ in inducing melanoma in genetically engineered mice in vivo. The results of both groups support a model in which the BRAF-specific inhibitors induce RAS-GTP-dependent RAF1 activation via the formation of BRAF-RAF heterodimers or RAF1 homodimers followed by recruitment of RAF1 to the plasma membrane, triggering the MEK-ERK pathway (Hatzivassiliou et al., 2010; Heidorn et al., 2010). In support of this mechanism, the investigators demonstrated co-immunoprecipitation of RAF1 with BRAF^WT^ after treatment with 885-A (Heidorn et al., 2010), or GDC-0879 (Hatzivassiliou et al., 2010), RAF1 kinase-domain homodimers when co-crystallized with GDC-0879 (Hatzivassiliou et al., 2010), and the translocation of BRAF and RAF1 to the plasma membrane accompanied by increased RAF1(S338) phosphorylation (Hatzivassiliou et al., 2010). In addition, the BRAF-inhibitors induced about 30% increase in the proliferation of established carcinoma cells (but not melanoma cell lines) derived from different tumors (Hatzivassiliou et al., 2010). However, as in our studies, both groups failed to detect BRAF-RAF1 heterodimers in response to PLX4720, a drug structurally related to PLX4032, and PLX4720 did not induce BRAF and RAF1 translocation to the membrane or an increase in RAF1(S338) phosphorylation (Hatzivassiliou et al., 2010). Furthermore, crystal structure revealed distinct differences in the mode of PLX4720 and GDC-0879 interaction with BRAF. While PLX4720 binding disrupts ion pairing, shifting the αC helix which inactivates the kinase, binding of GDC-0879 maintains the ion pair, orienting the αC-helix into an active conformation (Hatzivassiliou et al., 2010).

Altogether, although we have not uncovered the mechanism by which PLX4032 activated RAF1 in BRAF^WT^ melanoma cells, our data are consistent with the published reports and suggest that PLX4032 may have a different mode of action, which is independent of RAS-GTP because mutant RAF1 R89L that does not bind RAS was also activated to the same degree as its wild-type counterpart. It is also possible that PLX4032, like PLX4720, induces weak hetero- or homo- RAF dimers, as suggested by Marais and his collaborators that is not sustained through experimental manipulations.

In our hands, the presence of mutant NRAS in BRAF^WT^ melanoma cells was not required for PLX4032 induced ERK activation, cell detachment, loss of adherence and migration. However, so far, drug induced promotion of cell proliferation was observed in two NRAS mutant primary melanoma cell strains that did not acquire full independence from environmental growth stimuli. Therefore, dependency of growth response on NRAS mutation after treatment with PLX4032 should be further explored once growth factor-dependent BRAF^WT^/NRAS^WT^ melanoma cells become available. Altogether, our studies demonstrate persistence ERK activation in response to BRAF inhibition in BRAF^wt^ melanoma cells, the consequences on several downstream targets, the upregulation of a wide scope of ERK-responsive genes and the physiological changes that can confer growth advantage to these cells.

An important factor in cancer management is the ability to choose patients for specific therapy, monitor treatment and assess response. Very frequently, the presence of the activated target is not sufficient to assume responsiveness as shown for the activated/amplified EGFR where the levels of phosphorylated downstream mediators, Akt, ERK1/2 and Stat3 served as better markers (Emery et al., 2009). We demonstrated that melanoma cells heterozygous for V600E, but not V600K BRAF alleles were less sensitive to the inhibitory effect of PLX4032 and maybe be considered in patient selection and treatment. Our studies also suggest that monitoring the status of phospho-ERK1/2 and phospho-FAK S910 in tumor biopsies can be a good indicator for adverse effects, and that blood levels of IL8 and LIF may serve as markers, because normal cells were either inhibited or not-responsive to the drug.

## Materials and methods

### Cells

The melanoma cells listed in Table 1 were isolated from primary and metastatic lesions and the normal melanocytes from a giant nevus, all excised to improve patient quality of life. The specimens were collected with participants’ informed consent according to Health Insurance Portability and Accountability Act (HIPAA) regulations with Human Investigative Committee protocol. The melanoma cells from advanced lesions were grown in OptiMEM (Invitrogen, Carlsbad, CA, USA) supplemented with antibiotics and 5% fetal calf serum (basal medium). WW165 cells were cultured in basal medium supplemented with 3-isobutyl-1-methylxanthine (IBMX; Sigma-Aldrich, St Louis, MO, USA), and YULOVY, YUFULO and YUREEL-NV with recombinant fibroblast growth factor 2 (bFGF; Promega, Madison, WI, USA), IBMX and 12-*O*-tetradecanoylphorbol-13-acetate (TPA; Sigma), ingredients required for optimal proliferation (Böhm et al., 1995; Hoek et al., 2004). YUSIT1, 501 mel, YUGEN8 and WW165 were long-term cultures and the rest were early, short-term passage (passage 2–15). Keratinocytes were cultured from newborn foreskins in EpiLife medium (Cascade Biologics, Portland, OR, USA) and used during the first passage. BRAF, NRAS and PTEN mutations were identified by Sanger sequencing of gene-specific amplicons (Tables S1).

### Proliferation, migration and invasion assays

Cell proliferation was measured after 72-h incubation with increasing concentrations of PLX4032 (kindly provided by Plexxikon, Inc., Berkeley, CA, USA) employing the CellTiter-Glo® Luminescent Cell Viability Assay (Promega Corporation). The IC_50_ values (the dose that elicits 50% inhibition compared to vehicle control) were calculated from the slope of the drug response by linear interpolation. Soft agar colony formation was assessed after 10 days incubation with PLX4032 (1 μM) or DMSO (0.1%) by microscopical visualization. Soft agar cell viability was determined in 96 well plates with the CellTiter 96® AQueous One Solution Cell Proliferation Assay (MTS; Promega). Cell migration and invasion were evaluated with the CytoSelect™ 24-well kit (8 μm pore size, CBA-100-C; Cell Biolabs, Inc, San Diego, CA, USA). Migration was determined after 8 and 24 h incubations with PLX4032 (1 μM) or DMSO (0.1%). The number of cells that had migrated to the lower surface of the membrane was counted in three random fields or after colorimetric measurement at 570 nm.

### Western blotting and intracellular signaling

Melanoma cells were incubated with DMSO (0.1%) or PLX4032 (as indicated). Cell pellets were lysed in RIPA buffer supplemented with protease and phosphatase inhibitors, sonicated and centrifuged. Total cell extracts (16 μg protein/lane) estimated with the BioRad kit (Bio-Rad Laboratories, Hercules, CA, USA), were subjected to Western blot analyses (Halaban et al., 2009). The antibodies used were phospho-Mek1/2 pSer217/221, MEK1/2, phospho-Erk2 pThr202/Tyr204 (mAb), ERK1/2 (ERK 1/2, 137F5), phospho-p90RSK Ser380 (9D9 Rabbit mAb), phospho-p90RSK Thr359/Ser363, phospho-p90RSK Thr573, RSK1/RSK2/RSK3 (32D7), FAK (all from Cell Signaling Technology, Beverly, MA, USA), anti-actin (Sigma; mouse mAb), pFAK (S910; BioSource™, Invitrogen Corporation, Carlsbad, CA, USA, Cat # 44-596G), BRAF (goat AF3424; R&D Systems, Minneapolis, MN, USA), CREB-1 (24H4B; Santa Cruz Biotechnologies, Inc, Santa Cruz, CA, USA) and phospho-Ser133 CREB-1 rabbit polyclonal antibodies (20), and others described in Appendix S1. X-ray films were scanned and density of bands was assessed with ImageJ 1.42q (National Institutes of Health).

### Immune complex kinase assay

Melanoma cells were treated with PLX4032 (1 μM) or DMSO for the indicated period of time, collected by scraping on ice, washed with cold PBS containing phenylarsine oxide, lysed in NP40 (1%) or CHAPS (0.1%) lysis buffer supplemented with protease and phosphatase inhibitors, and PLX4032 (200 nM) for drug-treated cells. Antibodies against BRAF (H-145; Santa Cruz Biotechnology, Inc), RAF1 (#9422; Cell Signaling Technology), MEKK3 (C-term, EP600Y; Epitomics, Burlingame, CA, USA), and rabbit IgG (as a negative control) were pre-bound to Dynabeads®Protein G (Invitrogen). After three washes with lysis buffer, the beads were incubated with the cell lysates (200 μg proteins/assay, 100 μl final volume) for 30 min on a rotating wheel, and kinase activity was assessed on the bead-bound immune-complexes employing the Raf-1 Kinase Cascade Assay Kit (UPSTATE Cell Signaling Solutions, Millipore, Temecula, CA, USA) which measures the levels of [γ^32^P]-ATP incorporated into MBP. Kinase activities are expressed as pmol ATP incorporated into MBP/min after subtraction of control IgG values. Precipitated proteins were subsequently eluted with SDS sample buffer and analyzed by Western blotting with antibodies against BRAF (goat), RAF1 (mouse mAb, E-10, sc-7267; Santa Cruz Biotechnology, Inc), or MEKK3 (mouse mAb, Clone 40; BD Biosciences, San Jose, CA, USA).

### RAF1 suppression by siRNA

YUFIC melanoma cells were transfected with RAF1 siRNA (Smart pool siRNA; Dharmacon Inc; Chicago, IL, USA, Cat # L-087699-00), or Alexa Fluor 488 siRNA (100 nM/each) employing the DharmaFECT transfection reagent. At the end of 48 h, the cells were treated with PLX4032 or DMSO for 2 h, harvested and processed for Western blot analysis as above.

### Transfections

For kinase assays, YUFIC-BRAF^WT^ Melanoma cells were transiently transfected with plasmids encoding FLAG-tagged RAF1 or RAF1 R89L mutant that does not bind Ras (Dent et al., 1995; Laird et al., 1999) (kindly provided by Dr Deborah K. Morrison), using the Lipofectamine™ 2000 reagent (Invitrogen). Cells were treated with PLX4032 (1 μM) or DMSO for 16 h, after 32 h transfection, lysed in 0.1% CHAPS buffer, and anti-FLAG antibodies (M2; Sigma) and immune-complexes were tested for kinase activity as described above. The levels of immunoprecipitated proteins were assessed by Western blotting with anti-c-RAF (CST) antibodies. For RAF dimerization, melanoma cells (YUFIC-BRAF^WT^ and YULAC-BRAF^V600E)^) were co-transfected with FLAG-tagged RAF1 and Myc-His tagged RAF1 plasmids, harvested 2 days later after 12 h treatment with PLX4032 or DMSO, and cell lysates (200 μg/each) were subjected to immunoprecipitation with anti-Myc antibodies (c-Myc, sc-789; Santa Cruz Biotechnology, Inc) and Western blotting with anti-FLAG (M2 mAb) or anti-BRAF (goat) antibodies.

### Gene expression in response to PLX4032

YUDOSO-BRAF^WT^ cells were treated with PLX4032 (1 μM) for 8 and 24 h, or DMSO (24 h), RNA was extracted, processed for hybridization to NimbleGen arrays (A4542-00-01, design name HG18 60mer at Yale W. M. Keck microarray core facility and the results were subjected to bioinformatic analysis (Halaban et al., 2009). Changes in mRNA levels were validated by quantitative real-time RT-PCR employing ABI 7500 Real-Time PCR Systems (Applied Biosystems). *ACTB* expression was used as a reference to normalize for input cDNA. Gene specific primers are listed in Table S1.

### ELISA

IL8 levels secreted to the culture medium were measured after 24-h treatment with PLX4032 (1 μM) or DMSO with R&D DuoSet ELISA (#DY208).

## Supporting information

**Figure S1.** Expression of BRAF and RAF1 in melanomas.

**Figure S2.** Assessment of various survival pathways in response to PLX4032.

**Figure S3.** PLX4032 does not induce a RAF1 upstream target in BRAFWT melanoma cells.

**Figure S4.** Suppression of MEKK3 activity in response to PLX4032.

**Table S1.** Oligonucleotide primers pairs for PCR reactions.

**Table S2.** NimbleGen expression analysis.
