# Supplementary material for: PLX4032, a selective BRAFV600E kinase inhibitor, activates the ERK pathway and enhances cell migration and proliferation of BRAFWT melanoma cells
Source: Pigment Cell Melanoma Res. 2010 Feb 10;23(2):190–200. doi: 10.1111/j.1755-148X.2010.00685.x (PMC2848976; doi:10.1111/j.1755-148X.2010.00685.x)
Supplement: Supplementary file 1 [file pcr0023-0190-SD1.pdf]

Supplemental Materials and Methods

PLX4032, a Selective $BRAF^{V600E}$ Kinase Inhibitor, Activates the ERK Pathway and Enhances Cell Migration and Proliferation of $BRAF^{WT}$ Melanoma Cells

Ruth Halaban[1], Wengeng Zhang[1], Antonella Bacchiocchi[1], Elaine Cheng[1], Fabio Parisi[2], Stephan Ariyan[3], Michael Krauthammer[4], Jim McCusker[4], Yuval Kluger[2], and Mario Sznol[5]

Antibodies: The following list of antibodies were also used in our studies:

RAF1 (rabbit polyclonal), phospho-RAF1 Ser338 (56A6 Rabbit mAb), phospho-RAF1 Ser289/296/301 (rabbit polyclonal), phospho-RAF1 Ser259 (rabbit polyclonal), BRaf (L12G7, mouse mAb), phospho-Mek1/2 pSer217/221 (rabbit polyclonal), phospho p38 MAPK Thr180/Tyr182, c-Jun (60A8), Rabbit mAb; JNK (56G8, Rabbit mAb), phospho-SAPK/JNK Thr183/Tyr185 (Rabbit polyclonal), phospho-eIF4E Ser209, rabbit polyclonal), phospho-p70 S6 kinase Thr389 (108D2, Rabbit mAb), AKT (rabbit polyclonal), phospho-AKT Ser473 (D9E, Rabbit mAb),phospho-c-Jun (Ser63), Rabbit polyclonal, phospho-c-Jun (Ser73), Rabbit polyclonal; Phospho-Bcl-2 (Thr56), Rabbit polyclonal; Phospho-p53 (Ser15); Phospho-p53 (Ser20), Rabbit polyclonal Rabbit polyclonal; Phosphor-Bcl-2 (Ser70), Rabbit mAb; Phospho-MNK1 (Thr197/202); MKP5, #3483 Rabbit polyclonal all from Cell Signaling Technologies; Bcl2, Mouse mAb, from Abcam, Cambridge, MA; MEK kinase-1 (1-9C-2A, sc-449) and MKP-1, sc-1102 from Santa Cruz Biotechnologies

Supplemental Table S1: Oligonucleotide primers pairs for PCR reactions

Target	Primers	Product size (nt)
BRAF Exon 15	F1: 5’-CTACTGTTTTCCTTTACTTACTACACCTCAGA-3’<br>B1: 5' AACTCAGCAGCATCTCAGGGC-3'	210
NRAS exon 3	F1: 5'- CACACCCCCAGGATTCTTAC-3'<br>B1: 5'- TGGCAAATACACAGAGGAAGC-3'	150
PTEN mRNA	F1: 5'- TGCCATCTCTCTCCTCCTTTTTC -3'<br>B1: 5'- CCTCTGGTCCTGGTATGAAGAATG -3'<br><br>F2: 5'- GAGTAACTATTCCCAGTCAGAGGCG -3'<br>B2: 5'- CAAGTGTCAAAACCCTGTGGATG -3'	897
JUNB<br>RT-RTPCR	F1: AGTCCTTCCACCTCGACGTTT<br>B1: AATCGAGTCTGTTTCCAGCAGAA	
c-FOS	F1: 5'-CGAGCCCTTTGATGACTTCCT-3’<br>B1: 5'-GGAGCGGGCTGTCTCAGA-3’	

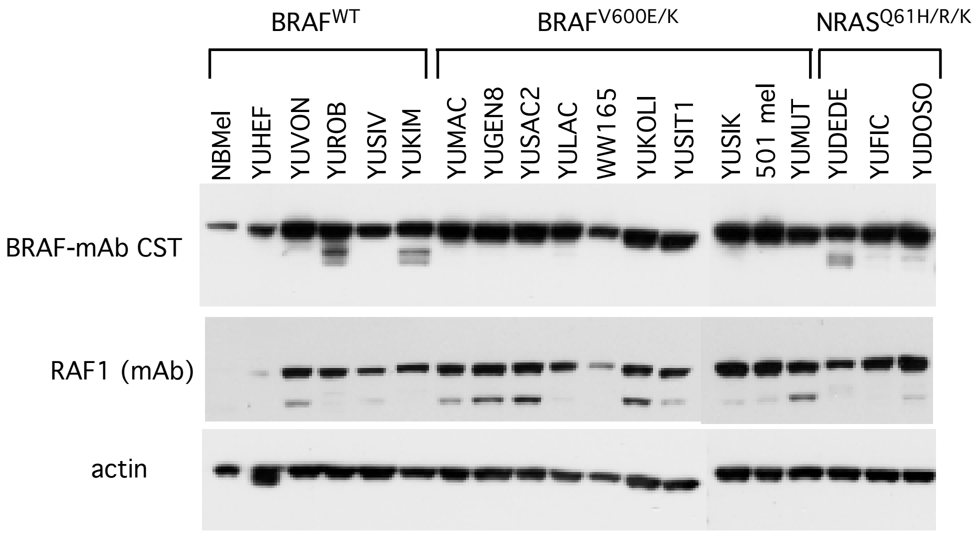

Supplemental Figure S1. Expression of BRAF and RAF1 in melanomas. Cell lysates from the melanoma cell strains used in our studies were subjected to Western blotting with anti-BRAF mAb (CST), RAF1 mAb, or actin as a control.

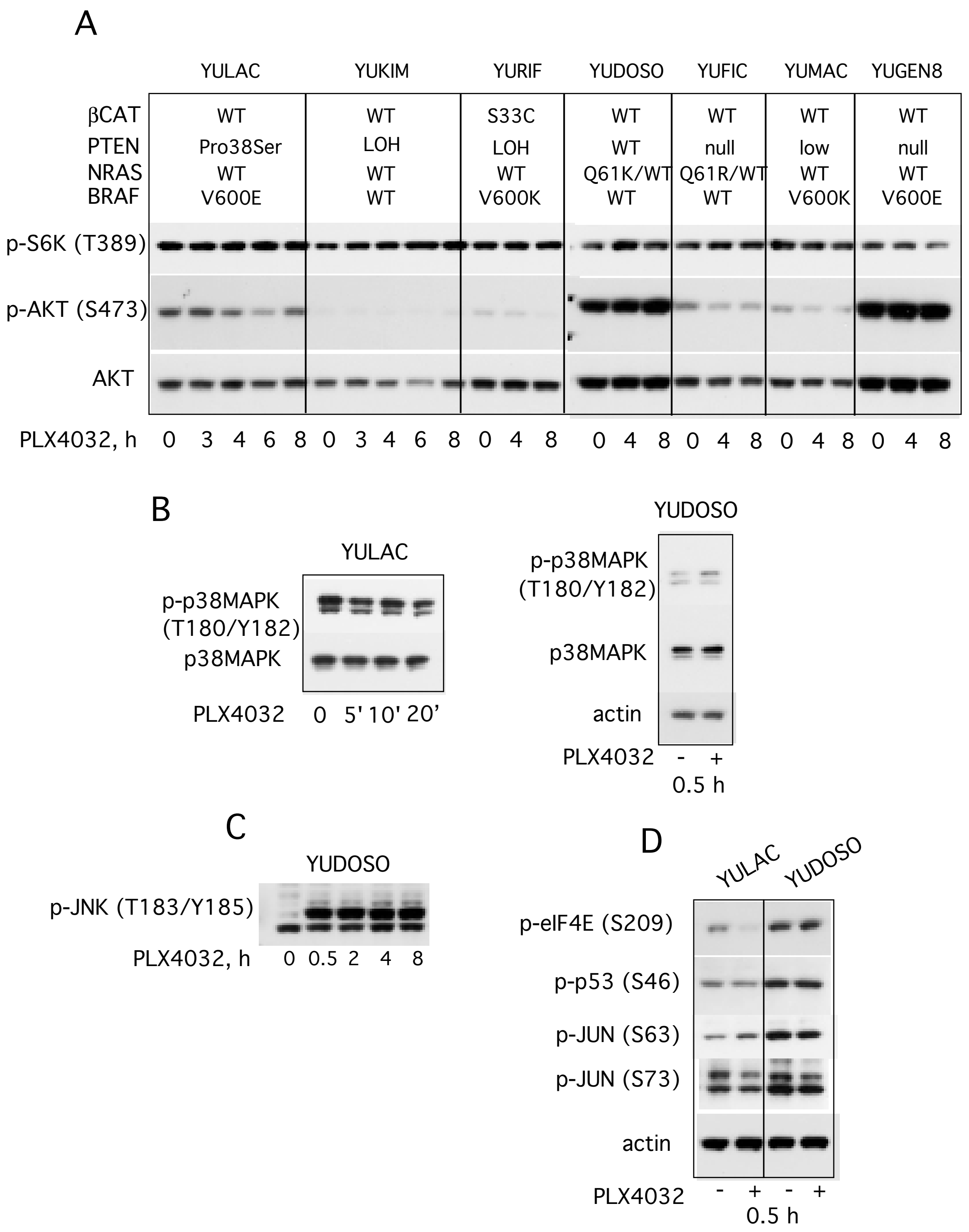

Supplemental Figure S2: Assessment of various survival pathways in response to PLX4032.
A, The AKT pathway. Western blots with phospho-S6K T389, phospho-AKT S473, AKT and actin as a loading control showing no increase in phospho-S6K or pAKT in response to PLX4032 (1 μM).
B, p38MAPK pathway. YULAC-BRAF^{V600E} and YUDOSO-BRAF^{WT} melanoma cells were treated with PLX4032 (1 μM) and harvested at the indicated time points. Cell lysates were subjected to Western blotting with anti-phospho p38MAPK (T180/Y182), or with anti p38MAPK showing little changes after treatment with PLX4032. C, JNK activation in YUDOSO-BRAF^{WT} by PLX4032 as revealed by probing with p-JNK (T183/Y185) antibodies. D, JNK downstream substrates eIF4E, p53, and JUN are not activated in YUDOSO-BRAF^{WT}. PLX4032 suppresses the JNK substrate eIF4E In YULAC-BRAF^{V600E} melanoma cells.

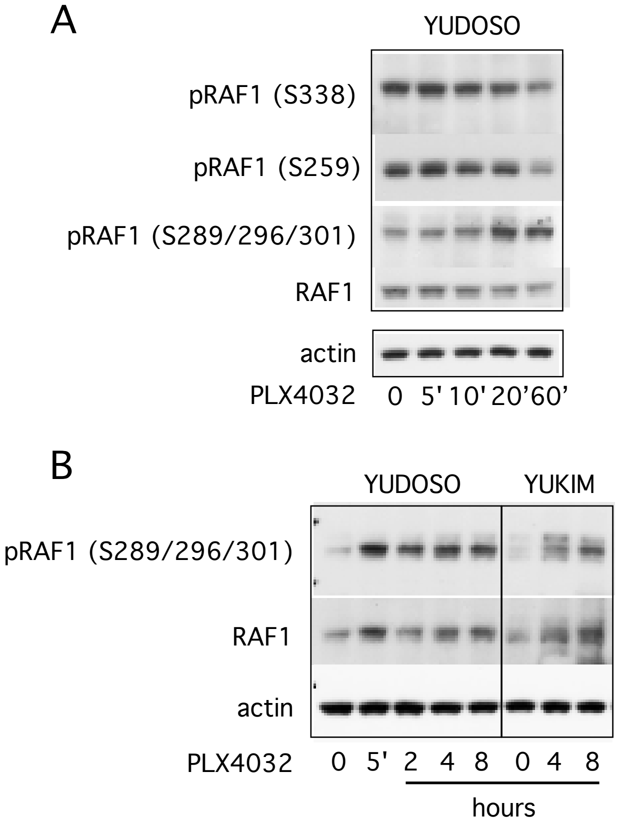

Supplemental Figure S3: PLX4032 does not induce a RAF1 upstream target in $BRAF^{WT}$ melanoma cells. A, and B, Western blots with antibodies to RAF1 or its phospho-isoforms (as indicated). In A, YUDOSO-$BRAF^{WT}$ melanoma cells were harvested after short treatments with PLX4032 (1 μM). The results show progressive decline in RAF1 activation domain phospho-Ser338 in YUDOSO-$BRAF^{WT}$ cells treated with PLX4032 probably dues to reduced levels of the protein in this fraction of the cell lysates. Likewise, the decline in phospho-Ser259, the inhibitory site, after 1 h treatmentmwith PLX4032 also correlated with reduced levels of total RAF1 protein (compare phospho-pRAF1 (S259)to RAF1). In contrast, there was a significant increase in phospho-RAF1 (Ser289/296/301), the MAPK phosphorylation sites that persisted in BRAF wild-type cells treated with the drug, shown in B.

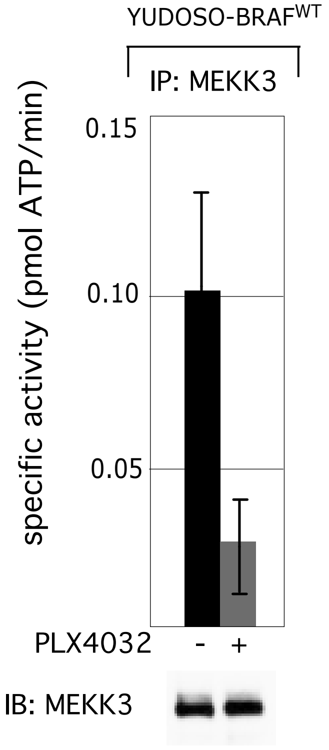

Supplemental Figure S4: Suppression of MEKK3 activity in response to PLX4032. YUDOSO-BRAF^{WT} melanoma cells were treated with PLX4032 (1 μM) for 1 h, harvested and MEKK3 immune-complexes were subjected to RAF1 kinase cascade assay in the absence of MEK as an intermediary. Kinase activity is expressed as picomoles [γ-^{32}P]-ATP incorporated into MBP. Each data point is an average of triplicate measurements ± STDV. The panels under each kinase assay shows Western blots of immunoprecipitated proteins eluted with SDS sample buffer and probed with antibodies to the MEKK3. There was no significant MEKK3 activity in YULAC-BRAF^{V600E} cells (data not shown), although equal amounts of protein were present in the immunoprecpitated material (data not shown).

UP	***Upregulated, persistent***
DP	***Downregulated, persistent***
UT	***Upregulated, temporary***
DT	***Downregulated, temporary***

Columns B,C,D log2(expression)
Columns E,F,G log2(fold_change)

A	B	C	D	E	F	G	H
SYMBOL	YUDOSO. DMSO	YUDOSO.P LX4032.8h	YUDOSO.P LX4032.24 h	YUDOSO. DMSO vs YUDOSO.P LX4032.8h	YUDOSO.P LX4032.8h vs YUDOSO.P LX4032.24 h	YUDOSO. DMSO vs YUDOSO.P LX4032[8h +24h]	CATEGOR Y
AATK	8.61	9.72	9.63	-1.11	0.09	-1.07	UP
ACPL2	11.55	12.60	12.85	-1.05	-0.25	-1.17	UP
ACTN2	9.44	10.46	10.51	-1.02	-0.05	-1.05	UP
AIM1	10.81	11.76	12.00	-0.95	-0.24	-1.07	UP
AIM2	9.58	10.88	11.26	-1.30	-0.38	-1.49	UP
AMOTL1	10.92	11.91	11.99	-0.99	-0.08	-1.03	UP
ARAP3	11.39	12.67	12.91	-1.28	-0.23	-1.40	UP
ARHGEF2	12.51	13.59	13.60	-1.08	-0.01	-1.08	UP
AVPI1	12.44	13.23	13.67	-0.80	-0.44	-1.02	UP
AXL	9.60	10.72	11.23	-1.12	-0.51	-1.37	UP
AXUD1	9.09	10.25	10.64	-1.16	-0.39	-1.36	UP
BCL2A1	12.08	13.18	13.28	-1.10	-0.10	-1.15	UP
C3orf32	10.56	11.78	12.06	-1.22	-0.27	-1.36	UP
C5orf47	7.80	9.46	9.48	-1.66	-0.02	-1.67	UP
CAPN5	9.37	10.50	10.86	-1.12	-0.37	-1.31	UP
CCR1	8.47	9.45	9.79	-0.98	-0.34	-1.15	UP
CD52	8.61	9.89	12.30	-1.28	-2.42	-2.49	UP
CD82	10.26	10.91	12.41	-0.66	-1.50	-1.41	UP
CDCP1	9.32	10.24	10.77	-0.92	-0.53	-1.18	UP
CDKN2B	10.10	11.25	11.82	-1.15	-0.57	-1.43	UP
CDKN2BAS	9.75	10.78	11.30	-1.03	-0.53	-1.29	UP
CLCF1	9.43	10.51	10.87	-1.08	-0.36	-1.26	UP
CLDN4	9.23	10.29	11.15	-1.07	-0.86	-1.49	UP
CLIP2	10.94	12.39	12.43	-1.46	-0.03	-1.48	UP
COL7A1	10.20	11.49	11.34	-1.28	0.14	-1.21	UP
COL8A1	9.78	10.46	11.11	-0.68	-0.65	-1.00	UP
CTGF	11.01	11.84	12.91	-0.83	-1.07	-1.37	UP
CTSG	9.54	10.13	11.34	-0.60	-1.21	-1.20	UP
DKK1	8.39	8.84	10.25	-0.45	-1.41	-1.15	UP
DUSP5	12.01	13.41	13.95	-1.40	-0.54	-1.67	UP
EGR1	12.42	14.41	14.40	-1.99	0.02	-1.98	UP
EGR2	10.07	11.13	11.51	-1.06	-0.37	-1.25	UP
EGR3	8.41	10.41	10.74	-1.99	-0.33	-2.16	UP
EHD2	11.24	11.98	12.68	-0.74	-0.70	-1.09	UP
ENTPD7	10.59	11.45	11.75	-0.86	-0.30	-1.01	UP
EPHB3	8.03	9.62	10.34	-1.60	-0.72	-1.96	UP
ERCC1	13.15	14.22	14.48	-1.08	-0.26	-1.21	UP
FAIM3	11.29	12.28	13.19	-0.99	-0.91	-1.44	UP
FAM19A3	8.32	9.00	10.19	-0.68	-1.19	-1.27	UP
FAM5B	7.73	8.47	9.51	-0.74	-1.04	-1.26	UP
FAM62C	8.21	9.02	9.46	-0.81	-0.44	-1.03	UP
FAM75A1	9.08	9.94	10.28	-0.85	-0.34	-1.03	UP
FAM75A2	9.08	9.94	10.28	-0.85	-0.34	-1.03	UP
FAM75A3	9.16	10.00	10.35	-0.84	-0.34	-1.01	UP
FAM75A5	8.98	9.81	10.26	-0.83	-0.45	-1.05	UP
FAM75A7	8.98	9.81	10.26	-0.83	-0.45	-1.05	UP
FBXO32	11.32	12.31	12.81	-0.99	-0.50	-1.24	UP
FCER1G	9.20	9.70	11.41	-0.50	-1.72	-1.36	UP
FHOD1	11.80	13.23	12.84	-1.43	0.38	-1.24	UP
FLJ46906	10.91	11.95	12.38	-1.04	-0.43	-1.26	UP
FOS	10.36	11.48	11.59	-1.11	-0.12	-1.17	UP
FOSB	10.22	11.65	11.58	-1.44	0.07	-1.40	UP
FRMD8	11.36	12.49	12.33	-1.13	0.16	-1.05	UP
GDF15	13.69	14.80	14.69	-1.11	0.11	-1.05	UP
GEM	9.76	12.26	12.68	-2.50	-0.42	-2.71	UP
GLDC	12.61	13.57	13.76	-0.95	-0.20	-1.05	UP
IL24	12.24	13.24	14.26	-1.00	-1.02	-1.50	UP
IL8	8.82	11.25	11.73	-2.43	-0.49	-2.67	UP
ISG20	8.91	9.99	10.33	-1.08	-0.34	-1.25	UP
ITGA3	11.99	13.54	14.01	-1.54	-0.47	-1.78	UP
ITGA5	11.81	13.04	13.49	-1.23	-0.45	-1.45	UP
ITGA7	8.88	9.79	10.17	-0.91	-0.38	-1.10	UP
ITGAX	8.43	9.16	10.90	-0.73	-1.74	-1.60	UP
ITGB3	12.24	13.11	13.40	-0.87	-0.29	-1.02	UP
ITPRIP	10.58	11.53	11.81	-0.95	-0.28	-1.09	UP
JUNB	10.95	12.07	12.26	-1.12	-0.19	-1.22	UP
KCNN4	9.93	11.70	12.40	-1.77	-0.70	-2.12	UP
LAD1	10.54	12.29	12.60	-1.75	-0.31	-1.90	UP
LAMP3	8.32	9.14	9.58	-0.82	-0.44	-1.04	UP
LIF	9.68	13.10	13.57	-3.41	-0.48	-3.65	UP
LOC100131	12.56	13.69	14.26	-1.13	-0.57	-1.41	UP
LOC100132	8.49	10.01	10.12	-1.52	-0.11	-1.57	UP
LOC100133	7.20	8.08	8.60	-0.88	-0.52	-1.14	UP
LOC440459	11.87	13.05	13.69	-1.18	-0.64	-1.50	UP
LUZP4	10.99	11.53	12.65	-0.54	-1.12	-1.10	UP
MDGA1	8.04	8.66	9.74	-0.62	-1.08	-1.16	UP
METTL7B	11.22	12.92	13.02	-1.71	-0.10	-1.76	UP
MFAP2	8.38	8.92	10.10	-0.54	-1.18	-1.13	UP
MICALL2	9.10	10.41	9.97	-1.31	0.45	-1.09	UP
MRGPRX1	10.35	12.16	12.33	-1.81	-0.17	-1.89	UP
MRGPRX3	10.88	12.69	12.80	-1.80	-0.11	-1.86	UP
MRGPRX4	11.53	13.26	13.56	-1.73	-0.30	-1.88	UP
MYEOV	9.13	10.78	11.80	-1.65	-1.02	-2.16	UP
NR4A1	10.66	11.72	12.49	-1.06	-0.77	-1.44	UP
OLFML3	9.92	11.66	13.13	-1.74	-1.46	-2.47	UP
P4HA2	11.51	12.36	12.96	-0.85	-0.60	-1.15	UP
PFKFB4	11.02	13.38	12.80	-2.36	0.58	-2.07	UP
PLAUR	11.31	12.76	13.13	-1.45	-0.37	-1.63	UP
PLEC1	10.89	12.14	11.94	-1.25	0.20	-1.15	UP
PLK3	11.27	12.54	12.86	-1.27	-0.32	-1.42	UP
PNLIPRP3	9.79	10.65	12.03	-0.86	-1.38	-1.55	UP
PPAP2B	11.90	13.06	13.01	-1.16	0.05	-1.13	UP
PTGES	8.53	9.29	10.18	-0.77	-0.88	-1.21	UP
PTPRH	10.13	11.17	11.42	-1.03	-0.25	-1.16	UP
RAC2	8.54	8.87	10.75	-0.33	-1.88	-1.27	UP
RDH5	10.76	12.28	13.26	-1.52	-0.98	-2.00	UP
RDH8	7.80	8.56	9.48	-0.76	-0.92	-1.22	UP
RHBDF2	10.31	11.19	11.92	-0.88	-0.73	-1.24	UP
S100A2	9.63	10.51	10.98	-0.88	-0.48	-1.12	UP
SCML4	10.17	11.50	11.73	-1.32	-0.23	-1.44	UP
SERPINB8	9.86	11.03	11.16	-1.17	-0.13	-1.24	UP
SERTAD1	9.46	10.54	10.58	-1.08	-0.04	-1.10	UP
SH2B3	10.35	11.54	11.43	-1.20	0.12	-1.14	UP
SIAE	8.78	9.74	10.29	-0.96	-0.55	-1.23	UP
SLAMF9	10.58	11.38	12.63	-0.80	-1.25	-1.43	UP
SLC16A6	12.26	13.07	13.77	-0.81	-0.70	-1.16	UP
SLC27A4	10.47	11.36	11.60	-0.88	-0.24	-1.01	UP
SLC2A14	9.77	10.43	11.12	-0.66	-0.69	-1.01	UP
SLC2A3	9.35	10.00	10.74	-0.65	-0.74	-1.02	UP
SLC6A6	9.93	10.92	11.19	-0.99	-0.28	-1.13	UP
SNCG	8.43	8.96	10.05	-0.53	-1.08	-1.07	UP
SPOCD1	8.33	9.71	10.18	-1.38	-0.47	-1.62	UP
SPP1	9.67	10.92	12.38	-1.25	-1.45	-1.98	UP
ST3GAL1	9.74	10.69	10.98	-0.95	-0.29	-1.09	UP
TBX3	10.12	11.14	11.27	-1.02	-0.13	-1.09	UP
TICAM1	11.11	12.13	12.57	-1.02	-0.44	-1.24	UP
TIE1	8.71	9.41	10.46	-0.71	-1.04	-1.23	UP
TM4SF19	13.29	14.48	14.27	-1.19	0.21	-1.09	UP
TM7SF4	9.15	11.59	12.29	-2.44	-0.70	-2.79	UP
TMEM158	8.84	9.72	10.08	-0.89	-0.36	-1.06	UP
TNFAIP3	9.07	10.15	10.41	-1.08	-0.25	-1.21	UP
TNFRSF12A	12.64	13.96	14.09	-1.32	-0.13	-1.38	UP
TNP1	7.96	8.72	9.23	-0.76	-0.51	-1.02	UP
TRAF1	10.32	12.22	12.47	-1.90	-0.26	-2.03	UP
TRIB1	12.39	13.30	13.55	-0.91	-0.25	-1.04	UP
TRPV2	11.00	11.93	12.12	-0.93	-0.19	-1.02	UP
TSPAN1	10.23	10.98	11.61	-0.75	-0.62	-1.07	UP
UCN2	11.86	14.61	14.39	-2.75	0.22	-2.64	UP
UPP1	12.82	14.19	14.22	-1.37	-0.02	-1.38	UP
USP53	10.98	11.99	12.58	-1.01	-0.59	-1.30	UP
VGF	12.43	13.78	14.28	-1.35	-0.49	-1.60	UP
ZYX	13.24	14.51	14.76	-1.27	-0.25	-1.39	UP
ANGPTL7	11.79	9.86	9.16	1.93	0.70	2.28	DP
ASPA	10.99	10.40	9.42	0.59	0.98	1.08	DP
C8orf46	10.89	9.70	9.90	1.19	-0.20	1.09	DP
FKSG83	9.20	7.92	7.87	1.28	0.05	1.31	DP
FLJ41603	9.38	8.23	8.31	1.15	-0.08	1.11	DP
HPGD	12.55	11.62	11.21	0.93	0.41	1.14	DP
LOC441956	12.32	11.27	11.01	1.04	0.27	1.18	DP
LRRTM4	11.28	10.33	10.02	0.96	0.30	1.11	DP
LSS	12.14	11.01	10.63	1.13	0.37	1.32	DP
MAL	11.50	10.59	9.31	0.90	1.29	1.55	DP
MAP2K6	11.16	9.64	9.65	1.52	-0.01	1.52	DP
PPP2R2B	12.72	11.35	11.17	1.37	0.18	1.45	DP
PRELP	11.44	10.66	9.15	0.78	1.51	1.54	DP
RBM24	8.73	7.67	7.71	1.06	-0.03	1.04	DP
SNAI2	13.19	12.28	12.07	0.91	0.21	1.02	DP
C1QTNF4	9.50	10.69	9.12	-1.19	1.57	-0.41	UT
CACNA2D2	8.03	9.04	8.15	-1.02	0.89	-0.57	UT
FAM9A	8.36	10.47	8.45	-2.11	2.02	-1.10	UT
GPR115	8.16	10.89	8.27	-2.73	2.62	-1.42	UT
IL4	9.08	10.08	8.86	-1.00	1.22	-0.39	UT
LOC147710	8.35	9.52	7.89	-1.17	1.62	-0.36	UT
LOC645781	8.52	11.87	8.30	-3.35	3.57	-1.57	UT
MC2R	7.69	8.72	7.78	-1.03	0.94	-0.56	UT
NMNAT2	8.37	9.38	8.34	-1.01	1.04	-0.49	UT
NPW	8.72	9.74	8.63	-1.02	1.11	-0.46	UT
NR2E1	9.60	10.66	9.26	-1.07	1.41	-0.36	UT
SSTR5	10.09	11.16	9.48	-1.08	1.68	-0.24	UT
SYNPO2L	8.40	9.85	8.47	-1.45	1.38	-0.76	UT
VIT	7.70	9.63	7.77	-1.93	1.85	-1.00	UT
SLITRK6	10.04	9.04	10.43	1.00	-1.39	0.31	DT
TXNIP	12.22	11.18	12.35	1.05	-1.18	0.46	DT

Supplementary Table S2: NimbleGen Expression Analysis. The log2 expression values for each array are shown, along with the log fold changes between DMSO and PLX4032.8h, PLX4032.8h and PLX4032.24h, as well as DMSO and PLX4032.[8h and 24]. The latter comparison merges the expression values of the two treatment time points (8h and 24h) with the geometric mean, and was used to identify genes with at least 2 fold persistent differential expression, i.e., expression changes that persist for 24 h after treatment (UP and DP in the table). We used the fold changes between DMSO and PLX4032.8h, and PLX4032.8h and PLX4032.24h to identify genes that show temporary fold changes, i.e. that would revert to baseline after 24h (UT and DT in the table). GSEA analysis: We used the GSEA program (http://www.broadinstitute.org/gsea/) to analyze our expression data for enriched pathway and motif gene sets. We uploaded the gct and cls files corresponding to our data, and set the Metric for ranking genes to Ratio_of_Classes, and the Permutation type to gene_set. All other parameters were set to default
